# Autoimmune congenital heart block: a case report and review of the literature related to pathogenesis and pregnancy management

**DOI:** 10.1186/s13075-023-03246-w

**Published:** 2024-01-02

**Authors:** Ying Huang, Jialin Deng, Jinghua Liu, Fangyuan Yang, Yi He

**Affiliations:** 1 Department of Rheumatology and Immunology, The Third Affiliated Hospital, Southern Medical University, No. 183, Zhongshan Avenue West, Tianhe District, Guangzhou, 510630 China; 2 Institute of Clinical Immunology, Academy of Orthopedics, Guangdong Province, Guangzhou, China; 3 Department of Pediatrics, The Third Affiliated Hospital, Southern Medical University, Guangzhou, China

**Keywords:** Anti-Ro/SSA antibody, Anti-La/SSB antibody, Autoimmune congenital heart block, Pregnancy management, Rheumatic diseases

## Abstract

Autoimmune congenital heart block (ACHB) is a passively acquired immune-mediated disease characterized by the presence of maternal antibodies against components of the Ro/SSA and La/SSB ribonucleoprotein complex that mainly affects the cardiac conducting system. ACHB occurs in 2% of women with positive anti-Ro/SSA and anti-La/SSB antibodies and causes a high risk of intrauterine fetal death, neonatal mortality, and long-term sequelae. In this review, we first describe a case of ACHB to provide preliminary knowledge. Then, we discuss the possible pathogenic mechanisms of ACHB; summarize the pregnancy management of patients with positive anti-Ro/SSA and anti-La/SSB antibodies and/or rheumatic diseases, the prevention of ACHB, and the treatment of ACHB fetuses; and propose routine screening of these antibodies for the general population. Careful follow-up, which consists of monitoring the fetal heart rate, is feasible and reassuring for pregnant women with positive anti-Ro/SSA and/or anti-La/SSB antibodies to lower the risk of ACHB in fetuses. Moreover, maternal administration of hydroxychloroquine may be useful in preventing ACHB in pregnant women with anti-Ro/SSA and/or anti-La/SSB antibodies.

## Introduction

Autoimmune congenital heart block (ACHB) is an acquired autoimmune disease characterized by dysfunction of the cardiac conducting system, resulting in partial or complete atrioventricular block. It develops in fetuses of women with anti-Ro/SSA and anti-La/SSB autoantibodies who may have autoimmune diseases, such as Sjögren’s syndrome (SS) and systemic lupus erythematosus (SLE); however, it may not be associated with other diseases. In addition, ACHB is detected most often between 18 and 24 weeks of gestation [1]. It has been reported that the morbidity of ACHB fetuses born to women with positive anti-Ro/SSA and/or anti-La/SSB autoantibodies is only 2% [2]. The recurrence rate is 12–25% in women who had a previous child with ACHB [3]. In addition, autoimmune congenital atrioventricular block occurs in approximately 1/20,000 live births, most of which may develop into 3° atrioventricular block from 1° or 2° atrioventricular block. The former is relatively rare but causes significant mortality [4]. In a large US-based registry of ACHB fetuses, the probability of death was 17.5%, and one third of these fetuses died in utero [5].

Herein, we report a case of ACHB and present a systematic review of the literature. This review is an attempt to emphasize the practical data and opinions on the pathogenesis of ACHB, particularly with respect to pregnancy management, prevention of ACHB, and treatment of ACHB fetuses.

## Method

To conduct this review, we searched the Web of Science and PubMed through 14 May 2023. Key search words were used that were relevant to autoimmune congenital heart block (“autoantibody-associated congenital heart block” OR “congenital heart block” OR “complete congenital heart block” OR “congenital complete atrioventricular block” OR “congenital heart disease”) and {mechanism (mechanism* OR effect) or pregnancy (pregnant OR pregnant* OR “gestation period”) or management/treatment (treatment* OR management* OR therapy)}.

Studies were included if they met the following eligibility criteria:

1. A quantitative empirical study published in a peer-reviewed journal in English
2. Investigated the potential underlying mechanisms of ACHB
3. Included management of pregnant women with positive anti-Ro/SSA and/or anti-La/SSB antibodies or rheumatic diseases during pregnancy
4. Included prevention and treatment of ACHB (standardized and validated therapies or individual items)

Studies were excluded if the full paper was not available upon request.

### Case report

A 39-year-old pregnant woman was admitted to the hospital for “amenorrhea for 37 weeks plus 5 days and fetal bradycardia for more than 8 weeks.” This was the patient’s third pregnancy, which was a natural pregnancy. The pregnant woman had delivered a live baby by cesarean section in November 2008 and terminated her second pregnancy at 40 days of gestation in 2009. She had no history of autoimmune diseases or heart diseases. At 13 weeks of pregnancy, the woman underwent prenatal routine examinations, including routine blood screening, liver function tests, renal function tests, syphilis test, and Down’s screening, and all the examination results were normal. On May 7, 2018, the amniocentesis results revealed alpha thalassemia without significant chromosomal abnormalities. On June 11, 2018, at 29 weeks of gestation, a fetal echocardiogram showed bradycardia of the fetus with a fetal heart rate of approximately 90 beats per minute, as shown in Table 1, and small accumulations of pericardial effusion. At 31 weeks of gestation, a repeat fetal echocardiogram revealed a fetal heart rate of 120 beats per minute. At 33 weeks of gestation, a new fetal echocardiogram was performed, which revealed a fetal heart rate of approximately 66 beats per minute, enlargement of the fetal cardiothoracic ratio, and small accumulations of pericardial effusion. At 37 weeks of gestation, the fetal echocardiogram showed fetal bradycardia with a heart rate between 35 and 43 beats per minute. Obstetric B-mode ultrasound showed an abnormal fetal heart rhythm and suggested possible abnormal heart development. The pregnant woman repeatedly refused further examinations and related treatments. On August 10, 2018, the woman delivered a baby girl via cesarean section. At this time, the baby’s heart rate was 42 beats per minute. The newborn had Apgar scores of 8 and 9 at 1 and 5 min, respectively. The echocardiogram suggested normal left ventricular function, moderate mitral regurgitation, and mild tricuspid and pulmonary regurgitation. High levels of anti-SSA/Ro antibodies (≥ 400.00 RU/mL, normal reference value: 0–20 RU/mL) and anti-Ro52 antibodies (301.77 RU/mL, normal reference value: 0–20 RU/mL) were detected in the serum of the mother, and increased levels of anti-SSA/Ro antibodies (403.00 RU/mL) and anti-Ro52 antibodies (200.72 RU/mL) were also detected in the neonate’s serum. The electrocardiogram revealed that the neonate had third-degree atrioventricular block. Doppler echocardiography indicated patent foramen ovale, moderate mitral regurgitation, and mild tricuspid and pulmonary regurgitation. The neonate was administered isoproterenol and epinephrine to improve heart rates, but it did not have a substantial effect. The parents refused the cardiologist’s recommendation of temporary pacemaker implantation for the neonate, and the neonate died on the second day of life. According to the clinical history, electrocardiogram and cardiac ultrasound results, and positive anti-Ro/SSA and anti-Ro52 antibodies, the neonate was diagnosed with autoimmune congenital complete atrioventricular block (CCAVB).

**Table 1:** Heart rate monitoring of the case during pregnancy

Date	Gestational weeks	Heart rate
28 April 2018	23^+1^ weeks	64 beats/min
11 June 2018	29^+3^ weeks	90 beats/min
22 June 2018	31 weeks	120 beats/min
6 July 2018	33 weeks	66 beats/min
8 August 2018	37^+4^ weeks	35–43 beats/min
10 August 2018	37^+6^ weeks	42 beats/min

## Pathogenesis of autoimmune congenital heart block

### Apoptosis, inflammation, and extensive fibrosis

A histological study of fetuses that died from ACHB revealed that apoptotic cardiomyocytes were extensively distributed in the fetal heart, especially in the sinoatrial node and atrioventricular node. Furthermore, calcification deposition, scar tissue, and macrophages coclustered with maternal antibodies in extensive fibrosis regions were observed. Macrophages were mostly located in the ventricular septum and thickened fibrous subendocardium region [6]. Human fetal cardiomyocytes are prone to apoptosis, promoting the translocation of SSA and SSB antigens to the surface [7]. During fetal physiologic development, apoptotic cardiomyocytes are cleared by resident cardiomyocytes. However, for patients with anti-Ro/SSA and/or anti-La/SSB, these antibodies may bind to SSA and SSB antigens, respectively, on the surface of apoptotic cardiomyocytes to form immune complexes. The formation of these immune complexes inhibits apoptotic cardiomyocyte physiological clearance. Afterwards, macrophages phagocytose apoptotic cardiomyocytes by binding Fcγ receptors to the immune complexes [8]. Then, the macrophages are activated to release proinflammatory factors such as TGF-β and TNF-α, which stimulate fibroblasts to differentiate into myofibroblasts, eventually leading to scarring [9, 10]. Taken together, the accumulation of apoptotic cardiomyocytes, inflammation, extensive fibrosis, calcification, and scarring play an important role in the induction of ACHB.

### Calcium channels’ dysregulation

L-type calcium channels, one of the three main classes of voltage-gated calcium channels, are targeted by calcium channel blockers. Four of the 10 *α*1 subunits, which include α1S, α1C, α1D, and α1F, form the pores of L-type calcium channels (LTCCs). LTCCs in human hearts include α1C calcium channels and α1D calcium channels [11, 12]. The α1C calcium channels are involved in the electrophysiological activity of the sinoatrial node in the human fetal heart, and α1D calcium channels are involved in the electrophysiological activity of the atrioventricular node [13]. T-type calcium channels in human hearts include α1G and α1H calcium channels, and the Ca current through α1G channels is involved in regulating cardiac impulse conduction through the atrioventricular nodes [14]. Several studies have indicated that anti-Ro/SSA antibodies bind to the α1G or α1D epitopes of cardiomyocytes and inhibit L-type and T-type calcium channels, suppressing the electrophysiological activity of cardiomyocytes in fetuses with ACHB [13, 15]. A recent paper has also addressed the interference role of anti-Ro/SSA antibodies on LTCCs in adults [12]. In particular, the presence of maternal p200-specific anti-Ro52 antibodies has been indicated to increase the risk of ACHB [16]. Utilizing a passive transfer model of ACHB, Ambrosi A and her colleagues [17] injected monoclonal antibodies into pregnant rats and discovered that only antibodies specific for the p200 domain of Ro52 induced ACHB, while antibodies targeting other domains of Ro52 did not. Additionally, p200-specific anti-Ro52 antibodies have been reported to promote intracellular calcium accumulation by recognizing calcium channels and reducing the contractility of cardiomyocytes, even inducing apoptosis [17, 18]. In addition, the activation of 5-HT4 receptors in human atrial cells induces cAMP-mediated activation of L-type calcium channels. Eftekhari et al. found that antibodies against residues 365–382 of the Ro52 peptide recognize and cross-react with residues 165 to 185 of the cardiac 5-HT4 receptor [19]. Then, anti-Ro52 antibodies inhibited the activation of serotonin-induced L-type calcium channels by blocking the activation of 5-HT4 receptors. Moreover, auxilin-deficient fetal mice have been reported to develop arrhythmia-like ACHB symptoms. Interestingly, compared with cardiomyocytes of wild-type mice, auxilin-deficient cardiomyocytes exhibit fewer α1D calcium channels on the cell surface [20]. Overall, anti-Ro/SSA antibodies may disturb the electrical activity of cardiomyocytes in the conduction system by dysregulating intracellular calcium homeostasis.

### Interferon

Numerous studies have suggested that type I IFN may contribute to the pathogenesis of ACHB. A clinical study of 9 women with ACHB pregnancies and 14 pregnant women with antibodies against Ro/SSA but without an ACHB complication found high expression of SIGLEC1 (a surrogate marker for the IFN signature that indicates cellular activation) and IFN-α in mothers of affected children [21]. Increased expression of IFN-regulated genes and plasma IFNα levels were detected in neonates exposed to anti-Ro/SSA and/or anti-La/SSB antibodies [22, 23]. In a recent study, researchers simulated cardiac injury conditions in vitro by incubating human fetal cardiac fibroblasts with supernatant from macrophages transfected with SSA/Ro60-associated ssRNA [24]. A transcriptome analysis of stimulated fibroblasts and healthy controls has provided clinical evidence for the upregulation of IFN-regulated genes in stimulated fetal fibroblasts and suggested that maternal autoantibody-induced cardiac damage may be secondary to the effect of type I IFN on fetal fibroblasts. Type I IFN is known to expand and activate NK cells, and a higher proportion of CD56^dim^CD16^hi^ NK cells has been found in cord blood from anti-Ro/La antibody-exposed neonates than in nonexposed controls [22, 25, 26]. NK cells may activate tissue-resident and infiltrating macrophages via IFN-γ and exaggerate apoptosis, promoting a local inflammatory reaction in the fetal heart. In addition, a single-cell transcriptome study from the heart of one fetus with ACHB has revealed a potential link between type I IFN and fibrosis [27]. Another concern is that IFN-α has been reported to increase the expression of Ro52 and induce apoptosis [28]. Several studies have indicated a potential direct arrhythmogenic effect of type I IFN [29–32].

### Other potential pathogeneses

ACHB may be related to viral infection. It has been reported that Ro/SSA antigens in ACHB fetal cardiomyocytes could translocate to the cell surface following cytomegalovirus infection [33]. The question of whether other viruses contribute to ACHB pathogenesis needs to be further illustrated.

## Management of pregnant women with positive anti-Ro/SSA and/or anti-La/SSB antibodies during pregnancy

### Hydroxychloroquine

Approximately 2% of fetuses from anti-Ro/SSA-positive mothers develop ACHB, which is extremely serious for the fetuses. Therefore, preventive treatments should be presented to these pregnant women. The 2020 American College of Rheumatology Reproductive Health Management Guidelines for Rheumatoid and Musculoskeletal Diseases conditionally recommend hydroxychloroquine (HCQ) treatment for all pregnant women with positive anti-Ro/SSA and/or anti-La/SSB antibodies to minimize the risk of ACHB [34]. In addition, it is recommended that pregnant women with a previous child with cardiac neonatal lupus syndrome (NLS) receive HCQ treatment [35]. Taking HCQ contributes to a lower risk of the current fetus developing ACHB. This may be because HCQ can inhibit the activation of Toll-like receptor (TLR) signaling and type I IFN [9, 21]. In a retrospective cohort study, 14 pregnant women took HCQ during pregnancy, while 48 pregnant women did not take HCQ [36]. Of the mothers taking HCQ throughout pregnancy, five approached 200 mg/day of HCQ for oral administration, while nine consumed 400 mg/day. One newborn in the HCQ group (7.1%) developed ACHB, while 7 newborns in the non-HCQ group developed ACHB (14.6%). The mother of the ACHB child in the HCQ group took HCQ again during her second pregnancy and gave birth to a healthy infant. A cohort study also observed that the incidence of ACHB fetuses in pregnant women taking HCQ (1/18, 5.5%) was much lower than that in the group not taking HCQ (6/21, 28.6%) [37]. These studies have shown that HCQ treatment possibly plays a vital role in reducing the prevalence of ACHB in fetuses.

### Intravenous immunoglobulin

Intravenous immunoglobulin (IVIG) can reduce transplacental autoantibody passage and increase the release of anti-inflammatory factors. However, whether IVIG treatment administered to the mother influences any outcome in ACHB is a current matter of debate. The risk of the fetus developing ACHB is approximately 2% in pregnant women with anti-Ro/SSA and/or anti-La/SSB autoantibodies, and the recurrence rate is 12–25% in women who had a previous child with ACHB [3]. In 2003, maternal administration of IVIG was initially proposed after a case series of 8 patients reported only 1 case of recurrent ACHB in mothers with a previously affected child [38]. However, in a small nonrandomized study, 20 pregnant women with positive anti-Ro/SSA antibodies who had a previous child with ACHB or neonatal lupus rash were administered 400 mg/kg IVIG every 3 weeks from 12 to 24 weeks of gestation, and 3 fetuses (3/20, 15%) were diagnosed with ACHB at the 19th, 20th, and 24th weeks of gestation [39]. The results suggest that low-dose IVIG does not decrease the recurrence of ACHB in high-risk pregnancies. Whether IVIG at higher doses would be more effective needs further study [40, 41].

### Daily ambulatory fetal heart rate monitoring and fetal ultrasound echocardiography

For pregnant women who have previously delivered infants with ACHB or NLS, the 2020 American College of Rheumatology Reproductive Health Management Guidelines for Rheumatology and Musculoskeletal Diseases recommend weekly fetal echocardiography, beginning at weeks 16–18 and continuing through week 26.

The recommendations conditionally recommend serial (less frequent than weekly; interval not determined) fetal echocardiography, beginning at weeks 16–18 and continuing through week 26 for pregnant women with anti-Ro/SSA and/or anti-La/SSB antibodies but no infant history of ACHB or NLS [34]. In fact, the transition from a normal rhythm to a third-degree atrioventricular block (AVB) occurs within 24 h, which highlights the importance of a closer surveillance of rhythm, eventually performed at home directly by the patients. It can prompt quicker access to dedicated care and improve outcomes at birth. In contrast, it is difficult for weekly monitoring to detect an early stage of ACHB before it progresses to third degree, and all data on therapies seem to emphasize that the earlier the stage at detection, the better the results of various therapies are. Daily ambulatory fetal heart rate monitoring (FHRM) allows for early detection of rhythm alterations and the administration of timely targeted treatments [42, 43].

## Pregnancy management in patients with rheumatic diseases

### Pregnancy management in patients with systemic lupus erythematosus

The 2020 American College of Rheumatology Guideline for the Management of Reproductive Health in Rheumatic and Musculoskeletal Diseases recommends that lupus patients should be screened for anti-Ro/SSA and/or anti-La/SSB antibodies before or during pregnancy. Moreover, given the titer and persistence of these antibodies, it is recommended that no repeated detection is needed during pregnancy [34]. According to the Canadian Rheumatology Association Recommendations for the Assessment and Monitoring of Systemic Lupus Erythematosus, anti-Ro/SSA and/or anti-La/SSB should be detected before pregnancy and during the first 3 months of pregnancy for women with SLE to monitor and implement prophylaxis treatment in a timely manner, mitigating the risk of gestating a fetus with ACHB and improving prognosis [44].

HCQ treatment should be recommended in pregnant SLE patients with anti-Ro/SSA antibodies to reduce the risk of fetuses with ACHB. A case–control study involving anti-Ro/SSA antibody-positive patients with SLE in pregnancy was published [45]. A pregnancy was considered exposed to HCQ if the patient took ≥ 200 mg/day during pregnancy, while a pregnancy was considered unexposed if HCQ was never taken or was discontinued after confirming pregnancy. Seven (14%) of the heart injury-related NLS children were exposed to HCQ compared with 56 (37%) of the controls (noncardiac neonatal lupus), indicating that exposure to HCQ reduces the risk of cardiac NLS.

### Pregnancy management in patients with Sjogren’s syndrome

Similarly, the British Society for Rheumatology guidelines for the management of adults with primary Sjögren’s syndrome suggest serial Doppler echocardiography to monitor atrioventricular time intervals during pregnancy in patients with Sjogren’s syndrome [46]. For all pregnant women with rheumatic diseases and positive anti-Ro/SSA and/or anti-La/SSB antibodies, HCQ treatment is conditionally recommended when they have low disease activity (Table 2) [34].

**Table 2:** Recommendations of different guidelines for pregnancy management

Guideline	HCQ	Fetal ultrasound echocardiography	Dexamethasone
2020 American College of Rheumatology Guideline for the Management of Reproductive Health in Rheumatic and Musculoskeletal Diseases [34]	Conditionally recommend treatment with HCQ during pregnancy	If no prior history of neonatal lupus, serial (interval uncertain) fetal echocardiography in weeks 16–26 If prior history of neonatal lupus, weekly fetal echocardiography in weeks 16–26	Abnormal fetal echocardiography: If first- or second-degree heart block, treat with dexamethasone 4 mg daily If isolated third-degree heart block (and no other cardiac inflammation), do not treat with dexamethasone
2017 The British Society for Rheumatology guideline for the management of adults with primary Sjogren’s syndrome [47]	HCQ may be continued throughout pregnancy and breastfeeding HCQ up to a maximum dose of 6 mg/kg is recommended for patients with pSS, especially those with skin and joint disease and fatigue. Patients should be monitored for evidence of a clinical and/or biological response (e.g., falling immunoglobulin levels) If no response after 12 months, consider stopping treatment	Monitor closely with serial ultrasound if anti-Ro and/or anti-La positive and consider referral to specialist center	/
2016 EULAR recommendations for women’s health and the management of family planning, assisted reproduction, pregnancy and menopause in patients with systemic lupus erythematosus and/or antiphospholipid syndrome [48]	/	Fetal echocardiography is recommended in cases of suspected fetal dysrhythmia or myocarditis, especially in patients with positive anti-Ro/SSA and/or anti-La/SSB antibodies	/

## Treatment for ACHB fetuses

### Fluorinated steroids

Fluorinated steroids may prevent the fetus from progressing from incomplete atrioventricular block to complete atrioventricular block [49]. Notably, for pregnant women with anti-Ro/SSA and/or anti-La/SSB antibodies and echocardiography showing fetal incomplete heart block, the 2020 American College of Rheumatology Guideline for the Management of Reproductive Health in Rheumatic and Musculoskeletal Diseases recommends oral dexamethasone of 4 mg per day [34]. However, the adverse effects of fluorinated steroids should not be ignored. Mothers are prone to suffer from hypertension, hyperglycemia, and excessive weight gain, while fetuses have a high risk for growth restriction, adrenal insufficiency, and oligohydramnios [50]. Whether dexamethasone prevents disease progression, reduces mortality, and avoids pacemaker implantation and cardiomyopathy in cases of second-degree and third-degree AVB is controversial [51, 52], but recent studies do not support its use [53].

### Plasmapheresis

Plasmapheresis may be a potential effective therapeutic strategy for ACHB fetuses. The efficacy of plasmapheresis in removing anti-Ro/SSA and anti-La/SSB antibodies was evaluated in 10 consecutive pregnant women with ACHB fetuses, and the degree of ACHB at detection and at delivery was recorded. In fact, 8 of the women showed a steady and significant decrease in anti-Ro/SSA and anti-La/SSB antibodies as the pregnancy progressed. Among them, the block reverted from a second to first degree in two fetuses and from second degree to sinus rhythm in the third fetus, indicating that plasmapheresis has beneficial effects on the reversal of incomplete atrioventricular block [54]. Regrettably, even receiving plasmapheresis, third-degree AVB in these fetuses seems to be permanent.

### Intravenous immunoglobulin

Regarding the effect of IVIG treatment on the improvement of ACHB, some case reports are promising. In a case report, the fetus of a mother with positive anti-Ro/La antibodies was diagnosed with 2:1 AV block and intermittent complete heart block at 28 weeks’ gestation [40]. The mother promptly received therapy with IVIG (400 mg/kg per day) for 5 days. Improvement in sinus rhythm with intermittent AV block was recorded throughout the remainder of the pregnancy. In a 10-year retrospective study of NLS in China, five babies who had a prolonged PR interval on ECG at birth were treated with intravenous immunoglobulin (IVIG) at a dose of 1 g/kg for 2 days, all of whom reverted to a normal sinus rhythm, providing evidence for the effectiveness of IVIG [55]. In a case series, 6 pregnant women with the fetuses diagnosed with second- and third-degree blocks were treated with a combination of IVIG 1 g/kg every 2 weeks, dexamethasone, and weekly plasmapheresis. Three of 3 cases of second-degree AVB reverted to normal sinus rhythm prior to delivery. All 3 cases with third-degree heart block remained stable prenatally, and only 1 needed pacemaker implantation at age 10 months [41]. This provided further support for the possible benefit of IVIG treatment in ACHB. However, further research is needed before definitive conclusions can be drawn regarding IVIG in ACHB treatment.

### Pacemaker implantation

**Fig. 1:**
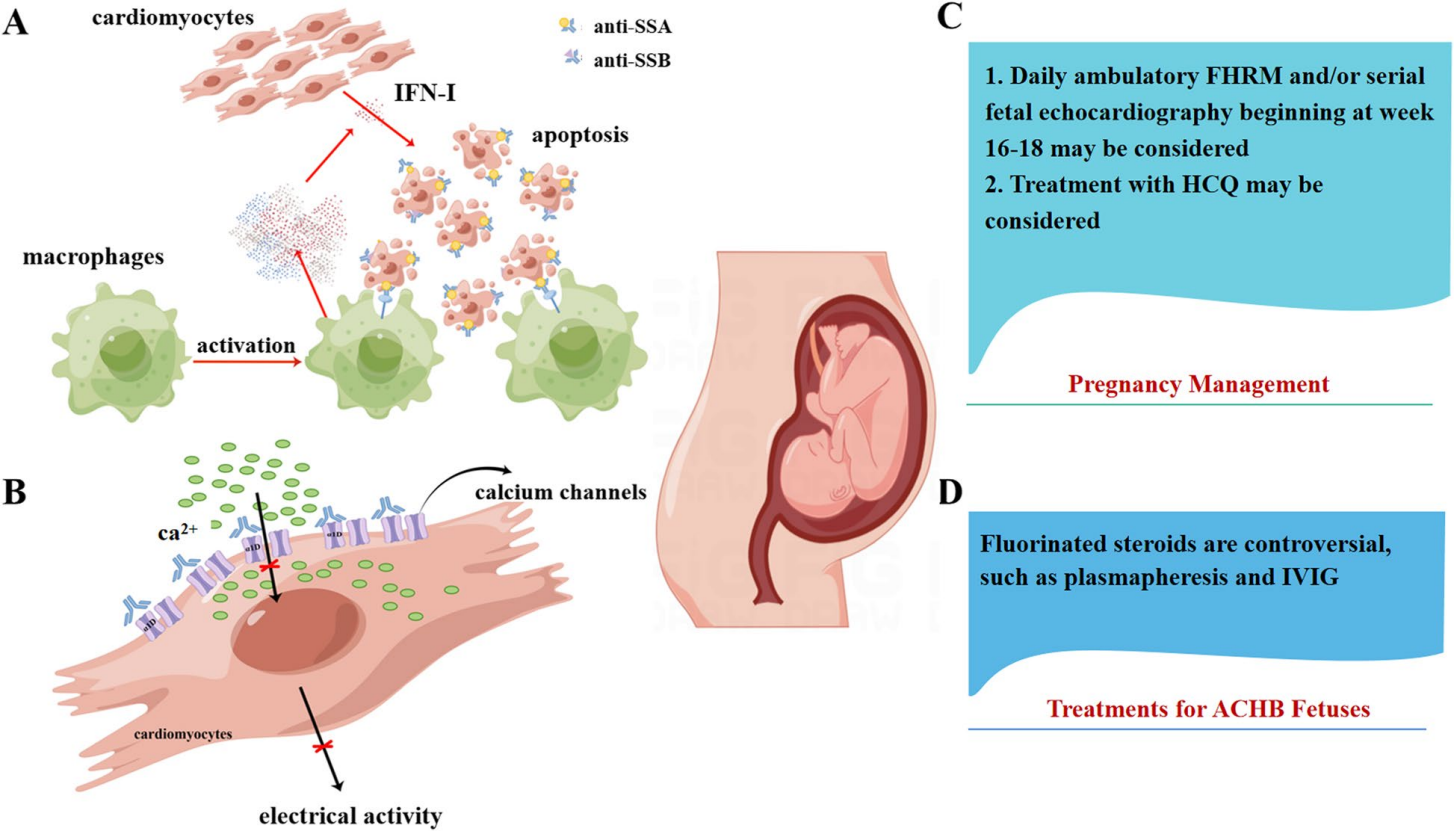
The potential pathogenesis of autoimmune congenital heart block and its prevention and treatment. **A** Accumulation of apoptotic cardiomyocytes, inflammation, extensive fibrosis, calcification, and scarring play an important role in the induction of ACHB. **B** Antibodies may disturb the electrical activity of cardiomyocytes in the conduction system through dysregulating intracellular calcium homeostasis. **C** Management in pregnant women with positive anti-Ro/SSA and/or anti-La/SSB antibodies or rheumatic diseases during pregnancy. **D** Treatment for ACHB fetuses

In a study of 16 ACHB fetuses diagnosed with third-degree block (12 diagnosed in utero and 4 at birth), 15 patients underwent pacemaker implantation during the first 2 weeks, and one patient underwent pacemaker implantation at 7 months [56]. During follow-up, all 15 patients had normal left ventricle (LV) function in the early postnatal period, and LV function significantly decreased in one patient. In a nationwide study of patients with complete atrioventricular block, all 127 patients underwent pacemaker implantation (median age at pacemaker implantation, 3.2 years) [57]. The survival rate was 96% at follow-up after approximately 9 years of pacing, indicating that pacemaker implantation may be a potential effective therapy for improving the survival rate of ACHB patients with complete atrioventricular block. However, pacemaker implantation has an adverse side effect that induces abnormal electrical activation patterns [58]. According to the American guidelines (2018) [59] and European guidelines (2013) [60], pacemaker implantation is recommended for ACHB fetus with third-degree block. In the European guidelines (2013), permanent pacing is indicated in symptomatic ACHB patients with third-degree block and is reasonable in asymptomatic patients. Figure 1 shows the potential pathogenesis of autoimmune congenital heart block and its prevention and treatment.

## Routine screening of anti-Ro/SSA and anti-La/SSB antibodies among women of childbearing age

Although ACHB in neonates born to mothers with positive anti-Ro/SSA and/or anti-La/SSB antibodies is uncommon, it can burden their lives and their families. A recent observational cohort study tested 7339 veterans for anti-Ro/SSA, 612 of whom were anti-Ro/SSA-positive (8.3%) [61]. Other studies have also reported the detection of anti-Ro/SSA (0.5–2.7%) in the general population [62–64]. Furthermore, a large observational study screened 2181 serum samples from the general population for disease-specific antinuclear antibodies (ANAs) and showed that the prevalence of anti-Ro/SSA in the general population was 2.7% (3.5% in females and 1.0% in males), and that of anti-Ro/SSB was 0.2% [64]. Therefore, it is important to screen for the antibodies described above in women of childbearing age. In addition, we can use predictive therapeutic strategies for these women to prevent the birth of ACHB fetuses.

## Conclusion

Autoimmune congenital heart block is rare, but it is associated with an increased risk of intrauterine fetal death, neonatal mortality, and long-term sequelae. To date, an increasing number of studies on the pathogenesis, prevention, and treatment of ACHB have been conducted. A deep understanding of its pathogenesis and knowledge of reasonable pregnancy management strategies and the latest therapeutic options are important to greatly reduce morbidity and mortality. The presence of anti-Ro/SSA and/or anti-La/SSB antibodies during pregnancy causes a significant risk for fetuses to develop ACHB. New evidence indicates the important role of type I interferon in the occurrence and development of ACHB. In addition, routine screening of anti-Ro/SSA and anti-La/SSB antibodies among women of childbearing age, daily ambulatory fetal heart rate monitoring, and monitoring of fetal echocardiography for pregnant women with positive anti-Ro/SSA and/or anti-La/SSB antibodies are feasible and reassuring methods to lower the risk of ACHB fetuses. Finally, prophylactic use of HCQ is important for pregnant women and is recommended in all women at risk for recurrence of CHB. Knowledge in this field of ACHB is continuously developing, and continuous updates are needed.

## Data Availability

Not applicable.

## Abbreviations

ACHB: Autoimmune congenital heart block
SS: Sjögren’s syndrome
SLE: Systemic lupus erythematosus
CCAVB: Congenital complete atrioventricular block
HCQ: Hydroxychloroquine
NLS: Neonatal lupus syndrome
TLR: Toll-like receptor
IVIG: Intravenous immunoglobulin
LV: Left ventricle
ANAs: Antinuclear antibodies
AVB: Atrioventricular block
FHRM: Fetal heart rate monitoring
